# A Visual Scan Analysis Protocol for Postural Assessment at School in Young Students

**DOI:** 10.3390/ijerph17082915

**Published:** 2020-04-23

**Authors:** Maria E. Alves, Daniel A. Marinho, Duarte N. Carneiro, Jorge Alves, Pedro Forte, Alan M. Nevill, Jorge E. Morais

**Affiliations:** 1 Department of Sports, Higher Institute of Educational Sciences of the Douro, 4560-708 Penafiel, Portugal; duartenuno.carneiro@gmail.com (D.N.C.); jorgealves1@me.com (J.A.); pedromiguelforte@gmail.com (P.F.); morais.jorgestrela@gmail.com (J.E.M.); 2 Research Center in Physical Activity, Health and Leisure (CIAFEL), Faculty of Sport, University of Porto, 4200-450 Porto, Portugal; 3 Department of Sports Sciences, University of Beira Interior, 6201-001 Covilhã, Portugal; marinho.d@gmail.com; 4 Research Center in Sports, Health and Human Development (CIDESD), 6201-001 Covilhã, Portugal; 5 Department of Sports Sciences, Instituto Politécnico de Bragança, 5300-253 Bragança, Portugal; 6 Faculty of Education, Health, and Wellbeing, University of Wolverhampton, Wolverhampton WV1 1LY, UK; a.m.nevill@wlv.ac.uk

**Keywords:** children, spine alignment, posture, X-ray, visual scan

## Abstract

The aim of this study was to compare the X-ray diagnosis with a non-invasive method for spine alignment assessment adopting a visual scan analysis with a plumb line and simetograph in middle-school students. The sample of this study was composed of 31 males and 50 females with an average age of 14.23 (± 3.11) years. The visual scan analysis was assessed at a school; whereas, the X-ray was performed in a hospital. The Wilcoxon signed-rank test was used to assess the differences between methods and scoliosis classifications (non-accentuated <10º and scoliosis >10º), and the Kappa was used to assess the agreement between methods. The comparisons between the methods revealed non-significant differences (z = −0.577; *p* = 0.564), with almost perfect agreement between tests (K = 0.821; *p* < 0.001). Moreover, no statistical significance was observed between methods by the scoliosis classification (z = −1.000; *p* = 0.317), with almost perfect agreement between tests (K = 0.888; *p* < 0.001). This research supports the conclusion that there are no significant differences between the two methods. Therefore, it should be highlighted that this field test should be used by physical education teachers in their classes, or in a school context, in order to determine misalignments or scoliosis prevalence among middle-school students.

## 1. Introduction

Physical education (PE) classes play an important role in child health, being considered as one of the most important contributors for health promotion in youngsters [1]. Even from a public health perspective, physical educators are encouraged to collaborate with public health professionals, doing periodic physical evaluations and developing physical education programs to promote child and adolescent health [2,3].

Posture is a public health concern due to its effect on musculoskeletal disorders [4]. Based on their wide range of activities, and mainly during their sitting time, children and adolescents are prone to adopting several improper postures both at home and school. This will promote muscular strength imbalances and new postural abnormalities [5]. Such anomalies may induce postural asymmetries that if not corrected, can result in painful syndromes in adult life. Posture is defined by the alignment of body segments at a specific moment [6]. The ideal posture is defined as the body in balance, aligned and with minimal possible energy expenditure. Body and postural misalignments may cause muscle tension and shortening, reducing the joints’ range of motion [7], which may result in musculoskeletal injuries and limit daily life activities [8].

One of the most prevalent pathologies in children and adolescents is low back pain (LBP) [9]. Additionally, one study recommended that different measures should be made to assess predisposing factors for youth back pain, such as scoliosis analysis. The authors recommend that different health professionals need to encourage people to perform several measures to manage back pain levels and prevent future adult back pain [10]. This pathology is frequently related with prolonged sitting postures, spine alignment, and abdominal muscle weakness [11,12]. Spine mechanical properties and asymmetrical load distribution may lead to scoliosis [13]. Both biomechanical and environmental factors seem to be involved in idiopathic scoliosis pathogenesis [13]. Indeed, the weight of school bags and individual anthropometric characteristics may contribute to LBP [14,15]. Children are also prone to adopting abnormal postures during their sitting time, which can also lead to postural asymmetries and hence future pathologies [16].

As children and adolescents spend a considerable amount of time at schools, it might be suggested that PE teachers may play an important role in detecting such postural concerns. They are responsible for physical activity lecturing and promotion, and consequently in detecting incorrect movements that may induce injuries. Eventually, children may not participate in practical classes due to LBP. Moreover, the early detection of postural misalignments by PE teachers may prevent muscle tension, shortening, and joints’ range of motion reduction in PE classes [7,8,9]. Thus, PE teachers might be aware of postural misalignments intending to avoid physical fitness decay, as well as LBP [7,8,9,17]. Moreover, such postural assessments at schools may quickly lead to medical referral and treatment, preventing future LBP [17]. Postural alignment can be assessed by a visual scan, photogrammetry, scoliometers, and X-ray, the latter being the gold standard method due to its applied technology [18]. However, PE teachers must deal with a set of implications in their analysis: (i) A simple and non-invasive method; (ii) easy to obtain immediate and accurate data during their classes; and (iii) safeguarding the children’s and adolescents’ interest due to ethical concerns (e.g., the use of pictures). A visual scan is a simple and non-invasive method that provides immediate feedback. The visual scan protocols usually include a simetograph to help evaluators classify subjects’ posture. This instrument is a translucent acrylic board, which is marked with a two-dimensional grid of 0.1-m divisions in both the width and length directions as references to detect postural misalignments [19,20].

Therefore, the aim of this study was to compare a simple and non-invasive method for spine alignment assessment by a visual scan with a plumb line and simetograph at schools (i.e., field test) with an X-ray diagnosis (i.e., clinical test—gold standard). It was hypothesized that non-significant differences would be found between the identification of the number of postural misalignment classifications using the X-ray and visual scan for postural assessment.

## 2. Materials and Methods

### 2.1. Participants

The sample of this study was composed of 31 males and 50 females with an average age of 13.40 ± 2.56 years. The males were 14.08 ± 2.97 years, with 1.64 ± 0.18 m of height, 55.68 ± 14.84 kg of body mass, and a body mass index of 19.38 ± 2.40 kg·m^−2^. Females were 13.00 ± 2.22 years, 1.54 ± 0.11 m of height, 45.62 ± 10.79 kg of body mass, and a body mass index of 18.94 ± 3.25 kg·m^−2^. Student’s evaluations were in their school, during the physical education classes. Written consents by parents or guardians were provided in order to take part in this study. All procedures were in accordance with the Helsinki Declaration regarding human research and the Institution Review Board approved the research (PROJ1.94/18).

### 2.2. Visual Scan Method (Field Test)

**Figure 1:**
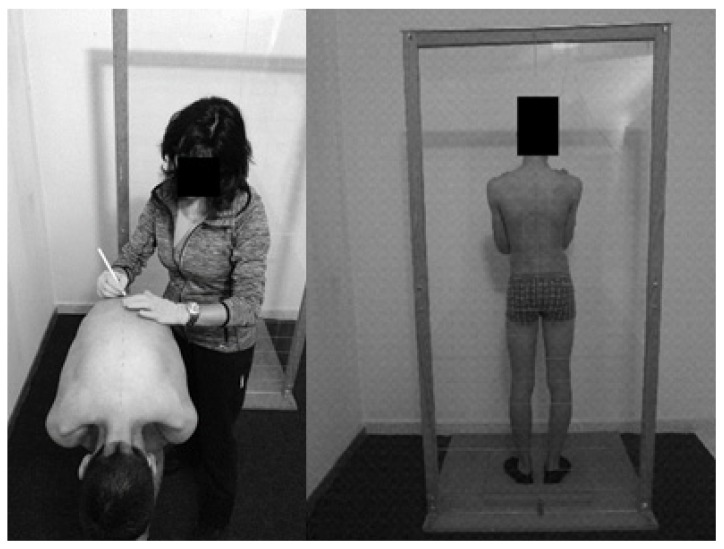
Left panel depicts the vertebrae marking process, the right panel depicts the visual evaluation.

The field test was conducted using an instrument designed and supported by the literature [20]. The instrument was based on a simetograph and adapted for this research. It consisted of an acrylic board supported by a wood base. On the base, there was a reference 0.6 m in length and 0.6 m in width for the foot position (with 30º of abduction) to avoid inter-student variability in foot position [21]. Each evaluated subject was positioned behind the transparent acrylic board with a 1.90 m height and 0.90 m width. In the upper part of the acrylic board was positioned a plumb line as a vertical reference. The acrylic board was marked with a two-dimensional grid using 0.1 m marked divisions in the vertical and horizontal directions. This instrument’s setup allowed the researcher to control the student’s overall position while performing the measurements. Indeed, this setup was used in both the field and clinical test to ensure a standardized measurement, and hence avoiding inter-student variability between tests. Students were placed in the orthostatic position, with both upper limbs crossed and hands touching the opposite shoulder (Figure 1, right panel).

**Figure 2:**
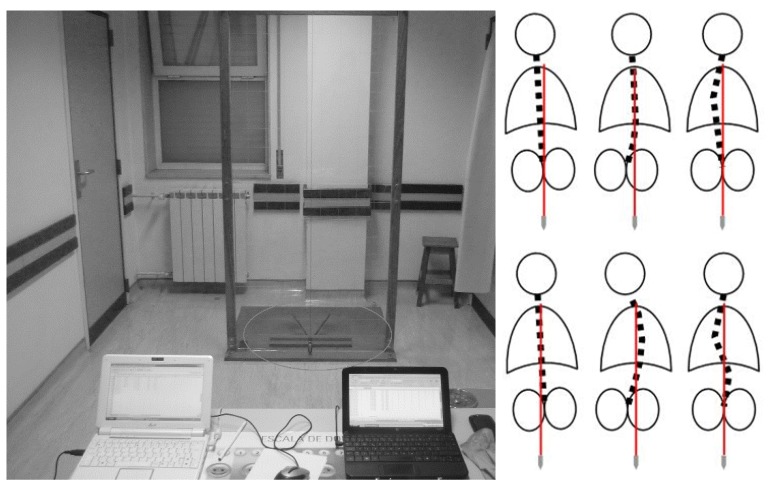
Left panel depicts the distance between evaluator and the instrument. The column’s left asymmetries are presented on the above right panel (equivalent for right misalignment) and bilateral misalignment, at right below panel.

Spinous processes were marked with demographic pencil after direct observation and palpation. The C7–L5 [22] vertebrae were marked with the participants in antero-posterior trunk flexion and the lower and upper limbs in the extension position (Figure 1, left panel). Each subject was as relaxed as possible to avoid possible lateral shoulders rotation, and fingers pointed to the ground with palm hands facing each other. Postural asymmetries were classified as: (i) Aligned and (ii) misaligned. The evaluator was seated at 2.5 m between the chair supports and the acrylic board base (Figure 2, left panel) to assess the participant’s posture on the posterior view of the frontal plane. The participants adopted an erect posture, looking forward, with flexed elbows pointing to the ground and the fingers touching the clavicles. Based on the previous visualization, the participants were also indicated according to their level of scoliosis. Scoliosis with more than 10º was considered as severe and with less than 10º non-accentuated [19]. Two trained evaluators performed the postural analysis. The inter-evaluator agreement revealed an almost perfect agreement (K = 1.00, *p* < 0.001; please report to the statistical analysis section) for both the alignment versus misalignment, and for the scoliosis degree visualization.

### 2.3. X-ray Method (Clinical Test)

**Figure 3:**
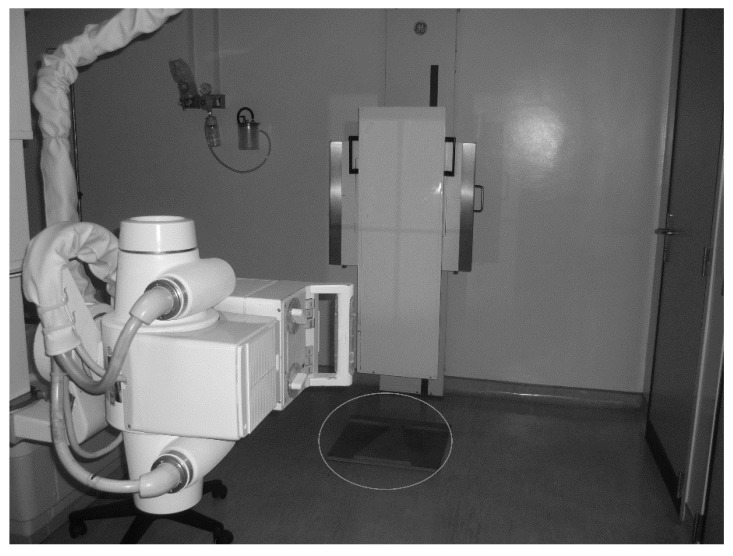
Philips X-ray machine with the wood base for the foot position.

After the visual scan assessment, the students were forwarded to a hospital for X-ray analysis. They were evaluated by a spine surgeon in a standing position for long radiographies, in the exact same position aforementioned, with the wood base for the foot position (Figure 3). A specialist medical doctor carried out blind evaluations with column radiographies with a Philips X-ray machine (Philips, System Medical - CYT9890, 010/87431 SN 14001512, Amsterdam). The evaluation permitted the assessment of neck, shoulders, scapulae, thoracic, and pelvic asymmetries.

### 2.4. Statistical Analysis

The Kolmogorov–Smirnov was used to test the normality of the data, where a non-normal distribution was observed. Cross-tabulation was used to understand the degree to which the two tests (i.e., field and X-ray) agreed and disagreed on their judgement of postural alignment or misalignment. The Wilcoxon signed-rank test was used to assess the difference between methods (*p* < 0.05). The Cohen’s Kappa was used to verify: (i) The inter-agreement between evaluators in the field test at schools and (ii) the agreement between the field and clinical test (aligned versus misaligned, and the level of scoliosis). It was interpreted as: (i) No agreement if K ≤ 0; (ii) none to slight if 0.01 < K ≤ 0.20; (iii) fair if 0.21 < K ≤ 0.40; (iv) moderate if 0.41 < K ≤ 0.60; (v) substantial if 0.61 < K ≤ 0.80; and (vi) almost perfect if 0.81 < K ≤ 1.00 [23].

## 3. Results

Table 1 presents the prevalence of alignment vs. misalignment for both tests. This sample was characterized by a substantial prevalence of misalignment (field test: n = 72, 88.9%; X-ray: n = 71, 87.7%).

Table 2 presents the cross-tabulation for postural agreements between the field and X-ray test. It was shown that the tests did not agree in only three cases.

The Wilcoxon signed-rank test between tests presented non-significant differences (z = −0.577; *p* = 0.564). Additionally, Cohen’s Kappa presented an almost perfect agreement between tests (K = 0.821; *p* < 0.001).

Table 3 presents the prevalence of non-accentuated scoliosis versus scoliosis for both tests. This sample was characterized by a small but higher prevalence of scoliosis (field test: n = 38, 52.80%; X-ray: n = 40, 55.60%).

Table 4 presents the cross-tabulation for the scoliosis-level agreements between the field and X-ray test. It was shown that the tests did not agree in only four cases.

The Wilcoxon signed-rank test between tests presented non-significant differences (z = −1.000; *p* = 0.317). Additionally, Cohen’s Kappa presented an almost perfect agreement between tests (K = 0.888; *p* < 0.001).

## 4. Discussion

This study aimed to compare a simple and non-invasive method for spine alignment assessment by a visual scan with a plumb line and simetograph at schools (i.e., field test) with an X-ray diagnosis (i.e., clinical test—gold standard). The pairwise comparison revealed non-significant (*p* < 0.05) differences between methods, and an almost perfect agreement was verified.

Several instruments are used for postural assessment and detection of scoliosis [22,24]. The X-ray, computed tomography, and the magnetic resonance methods are gold standard methods to detect spine structural, neurologic, and congenital problems [25]. However, magnetic resonance is a highly expensive procedure, and hence, it is not commonly used by physicians. Computed tomography and X-ray methods expose patients to radiation, and hence compromise children´s health [26]. In this sense, due to the high-cost methods and to avoid radiation exposure, it is suggested that there is a need for alternative tools/instruments for children’s postural assessment, and consequently scoliosis detection [27]. Moreover, it can be claimed that PE teachers could play a major role in detecting misalignments or even scoliosis in children and adolescents in an early stage [17]. The field test (photogrammetry, scoliometer, and visual scan methods) does not expose participants to radiation. However, the visual scan analysis allows immediate and accurate results [19,20] to be obtained and safeguards children and adolescent health (i.e., the use of pictures) [24,28,29]. Unfortunately, scoliometers are not pedagogical instruments and many schools are not equipped with them.

Studies have suggested photogrammetry methods (digital image-based postural assessment) to measure posture [24,28]. Such methods are low cost and non-invasive. However, postural assessment by photogrammetry is a time-consuming method that requires a set of procedures to calibrate and analyze the images [29]. Thus, photogrammetry may take too much time to assess large samples of participants. Upon this, pictures would have to be stored and ethical concerns would be raised. Among the different methods, the visual scan is considered as the most commonly used method for a preliminary analysis [30]. Some visual scan methodologies for postural assessment include a plumbline as a vertical reference, or a simetograph [31]. Our study revealed non-significant differences, with an almost perfect agreement between methods (i.e., field test versus clinical test) for the alignment and scoliosis measurement.

Techniques, such as palpation, simetograph, plumbline, and vertebrae landmarks, were used to assess scoliosis, which appear to be a good replacement for X-ray [32]. The almost perfect agreement between methods could be explained by such a cluster of methods for scoliosis analysis. Palpations are a useful technique for the identification of scoliosis, and simetograph is considered a valid tool for postural assessment, reducing the evaluator error [33]. Assessing the non-accentuated (< 10º) and scoliosis cases (> 10º), a non-significant difference with an almost perfect agreement (K = 0.888; *p* < 0.001) was also found between methods. Again, this might be explained by the aforementioned cluster of methods for scoliosis analysis (palpation, simetograph, plumbline, and landmarks). It should be highlighted that several studies have analyzed posture, based on the techniques aforementioned (i.e., photogrammetry, visual scan, etc.) [24,28]. By contrast, few studies have assessed misalignments based on X-ray [34], enabling researchers to compare visual scan techniques with a gold standard method, such as the X-ray. However, in our study, the false positives were 2.2% and the false negatives were 1.1%. This indicates that use of the instrument is sensible to assess youth’s postural misalignments. Nearly 2.2% of the subjects classified with misalignment were aligned and only 1.1% classified as aligned were misaligned.

Overall, this non-invasive method (i.e., field test) was shown to be a valid and reliable postural assessment in young students, in a school context. Moreover, these methods allowed the assessment of non-accentuated and severe (scoliosis) cases. This is a less time-consuming field test, which PE teachers may use to assess young students’ postural misalignments without costs, and to gather accurate and valid data. Moreover, they might be able to adjust PE classes based on misalignments’ severity to lecture classes focusing on LBP rehabilitation or injury prevention. As a main limitation, it can be considered that the postural behaviors were not controlled. Nonetheless, the test agreement was almost perfect (with non-significant differences) regardless of the presentation of misalignments. Further studies are recommended in adult populations and longitudinal analysis may help to predict postural changes in youth as they grow into adults.

## 5. Conclusions

It can be concluded that there are non-significant differences (with an almost perfect agreement) between the two methods (i.e., field vs. clinical), in both alignment vs. misalignment and non-accentuated vs. accentuated scoliosis. Therefore, we encourage PE teachers to adopt the field test when assessing postural asymmetries in young students. Moreover, this analysis should be performed based on a preventive role in order to quickly detect posture misalignments.

## Figures and Tables

**Table 1:** List of the articles selected for analysis, including the article aim and sample, as well as the sensor specifications.

Postural Diagnosis	Field Test	X-ray
Alignment	9 (11.1%)	10 (12.3%)
Misalignment	72 (88.9%)	71 (87.7%)

**Table 2:** Cross-tabulation for the postural assessment.

		X-ray		
		Misalignment	Alignment	Total
**Field test**	Alignment	2	70	72
	Misalignment	8	1	9
	Total	10	71	81

**Table 3:** Frequency and percentage of non-accentuated scoliosis versus scoliosis in the field and X-ray tests.

Scoliosis Level	Field Test	X-ray
Non-accentuated	34 (47.20%)	32 (44.40%)
Scoliosis	38 (52.80%)	40 (55.60%)

**Table 4:** Cross-tabulation for the scoliosis assessment.

		X-ray		
		Non-accentuated	Scoliosis	Total
**Field test**	Non-accentuated	31	3	34
	Scoliosis	1	37	38
	Total	32	40	81
